# Comparison of skin prick test and prick‐to‐prick test with fruits and vegetables in the diagnosis of food allergy

**DOI:** 10.1002/clt2.12375

**Published:** 2024-07-05

**Authors:** Severina Terlouw, Frank E. van Boven, Monika Borsboom‐van Zonneveld, Tineke de Graaf‐in ’t Veld, Roy Gerth van Wijk, Paul L. A. van Daele, Maurits S. van Maaren, Jac H. S. A. M. Kuijpers, Sharon Veenbergen, Nicolette W. de Jong

**Affiliations:** ^1^ Department of Allergology Albert Schweitzer Hospital Zwijndrecht The Netherlands; ^2^ Internal Medicine Allergology & Clinical Immunology Erasmus MC University Medical Center Rotterdam Rotterdam The Netherlands; ^3^ Department of Immunology Laboratory Medical Immunology Erasmus MC University Medical Center Rotterdam Rotterdam The Netherlands

**Keywords:** diagnosis, food allergy, homemade extracts, prick‐to‐prick test, skin prick test

## Abstract

**Introduction:**

Prick‐to‐prick (PTP) test with fresh food is accepted as a reliable tool for measuring sensitization to fruits and vegetables. Not all fruits and vegetables are available throughout the year. The objective of this study was to investigate whether skin prick test (SPT) performed with frozen juice of fruits and vegetables (FJFV) is a good alternative to PTP tests performed with fresh fruits and vegetables (FFV).

**Methods:**

Adult patients suspected of having a food allergy to fruits and/or vegetables were included. A questionnaire was used to score symptoms after consumption of apple, kiwi, peach, tomato, and carrot. SPTs with FJFV, and PTP tests with FFV were performed. Intra‐class correlation coefficients (ICC) between the SPT and PTP test results were calculated. The sensitivity and specificity of both diagnostic tests towards food allergen specific symptoms (FASS) were calculated.

**Results:**

Thirty‐six patients were included. FASS was positive in 75% for apple, 53% for kiwi, 44% for peach, 25% for tomato, and 22% for carrot. ICC between SPT and PTP test results were moderate for apple (0.72) and kiwi (0.71), strong for peach (0.75) and tomato (0.89), and very strong for carrot (0.94). Sensitivity was equal for the SPT and PTP tests for apple (0.93), peach (0.81), and carrot (1.00), and comparable for kiwi (0.50 resp. 0.70), and tomato (0.44 resp. 0.56). Specificity was equal for apple (0.33), peach (0.15), and carrot (0.41), and comparable for kiwi (0.29 resp. 0.21) and tomato (0.80 resp. 0.72).

**Conclusions:**

Results of SPT with FJFV and PTP test with FFV are comparable. SPT with FJFV is a good alternative in the daily practice of the allergists.

## 1 INTRODUCTION

Oral allergy syndrome (OAS) to fruits and/or vegetables is the most frequent food allergy reported in adult patients in Europe.^1^, ^2^ The reported reactions triggered by consumption of fresh fruits or raw vegetables are attributed to cross‐reacting homologous proteins found in foods and pollens.^3^ According to Beyer et al., the fruits and vegetables that most frequently elicit allergic reactions in pollen allergic patients are apple (78%), carrot (52%), peach (49%), kiwi (41%), and tomato (23%).^4^ Diagnosing a suspected food allergy accurately is of great importance, first to prevent severe allergic reactions in patients after ingestion of the causative food, and second to avoid unnecessary dietary limitations, resulting in a potential insufficient diet. The cornerstone of the diagnosis of food allergy is the clinical history, complemented by a combination of results of allergy tests such as skin prick test (SPT), with Double Blind Placebo Controlled Food Challenges (DBPCFC) being the gold standard.^5^ The negative predictive value (NPV) of SPTs often reaches 90% or more.^6^ The SPT is minimally invasive, safe, cheap, and affords evidence to diagnose sensitization of a suspected type I food allergy.^2^, ^7^ The test results are available within 15 min. In an EAACI position paper by Klimek et al. on in‐vivo diagnostic test allergens in Europe, the importance of reliable allergens used for SPT was stressed.^8^ In a more recent published guideline, Santos et al. provided recommendations for diagnosing IgE‐mediated food allergy using the Grading of Recommendations, Assessment, Development, and Evaluations (GRADE) approach.^9^ The quality of the allergen extracts used in SPT influences the results, and thus the diagnosis. Some fresh food allergens lose their allergenic properties rapidly during the extraction process.^10^ Furthermore, not all fruits and vegetables can be bought throughout the whole year. Corresponding commercially available extracts of fruit and vegetables have a low allergenic activity; the proteins are destroyed during the manufacturing process of the extracts.^10^, ^11^ Frozen aliquots of fresh fruits and vegetables, defrosted and individually stirred with a disposable plastic Pasteur pipette shortly before use in SPT, would be a good solution to perform SPTs at any time.^1^ The reproducibility and stability of homemade food allergen extracts of coriander, hazelnut, peach, and sesame seed were studied by de Jong, and no significant differences between fresh food applied by PTP tests, and 3 and 6 months stored by −20°C extracts applied by SPT were found.^12^ The aim of this study was to investigate whether SPT performed with frozen juice of fruits and vegetables (FJFV) is a good alternative for PTP tests performed with fresh fruit and vegetables (FFV). We chose to investigate the SPT and PTP test results of apple, kiwi, peach, tomato, and carrot. These foods are widely consumed and have been increasingly reported as causes of allergic reactions, particularly in adults with pollinosis.^13^

## 2 MATERIALS AND METHODS

### 2.1 Study population

Adult patients with allergy specific symptoms after ingestion of fruit and/or vegetables in the clinical history and visiting the outpatient clinic at the Department of Allergology at the Albert Schweitzer hospital, were asked to participate in the study. All participants stopped their anti‐histamines for at least 72 h before the SPT. Medical ethical approval was obtained for this study on 27 December 2022, trial number MEC‐2022‐0716, NL81413.078.22. After patients were able to ask questions and sign informed consent, the inclusion took place from August 2023 until December 2023. Inhalant allergies and concomitant medication used were reported. A food allergy‐focused clinical diet history questionnaire was used to score symptoms after consumption of apple, kiwi, peach, tomato and carrot. Symptoms were defined as the incidence of oral itching, with or without angioedema of the lips and/or tongue (OAS), gastrointestinal symptoms (GI), skin symptoms, and/or respiratory symptoms (respiratory). The respective food allergen specific symptoms (FASS) questionnaire used is included online.

### 2.2 Skin prick tests and prick‐to‐prick tests

All patients underwent a SPT and PTP tests with apple, kiwi, peach, tomato, and carrot. The difference between SPT and PTP is that in the PTP test, the needle is first pricked into the fresh, intact, and unpeeled fruit/vegetable, near the stalk. Subsequently, the juice sticking to this needle is transferred into the skin of the participant.^5^ The SPT was performed by applying a drop of defrosted and mixed FJFV to the skin of the volar aspect of the forearm. SPT and PTP tests were conducted at the same time, on the volar surface of the forearm. Subsequently, in both tests, the dermis was punctured with a disposable standardized skin test needle (ALK‐Abelló, Copenhagen, Denmark), as recommended in the established EAACI guidelines.^8^ Dilution buffer (ALK‐Abelló, Copenhagen, Denmark) was used as a negative control and histamine dihydrochloride 10 mg/mL (ALK‐Abelló, Copenhagen, Denmark) as a positive control. To avoid technical bias, the same study nurse performed all SPTs and PTP tests. Skin test results were obtained after 15 min; the contours of the allergen‐induced wheal were encircled with a fine‐tip pen and transferred to a record sheet by means of a translucent tape (ALK‐Abelló, Copenhagen, Denmark). For quantitative analysis, we compared the Histamine Equivalent Prick results (HEP/PAAMOST)^14^, ^15^ of the SPT and PTP tests for the distinct food allergens. In addition to HEP measurements, the allergen induced mean wheal diameter was measured to decide on a positive or negative skin test result (positive ≥3 mmØ) according to the EAACI international guidelines.^7^

### 2.3 Homemade food allergen extracts

The raw material for each tested food allergen extract was carefully screened to select the material that best represented the allergen. For PTP tests, ripe fruits and vegetables were bought fresh every week at the local fruit and vegetable shop, and stored separately at 4°C. For both SPT and PTP tests, apple (Golden Delicious), kiwi (Hayward Green), peach (Royal Summer), tomato (Princess), and carrot (*Daucus carota*) were washed with tap water. For SPTs, each food was processed with peel and homogenized in a food processor. Juice was immediately stored in small portions for single use at −20°C.^15^, ^16^ All portions were prepared within 2 min and stored immediately, to avoid bias in processing the material.

### 2.4 Serum‐specific IgE

Serum‐specific immunoglobulin E (sIgE) levels of 35 participants were evaluated with the Allergy Explorer (ALEX) multiplex array (Macro Array Diagnostics, Vienna, Austria). The result was considered positive when >0.3 kUA/L.

### 2.5 Statistical analysis

Comparison of the HEP results of the SPT and PTP tests was done by calculating the intra‐class correlation coefficient (Intra‐class correlation coefficients (ICC)) between the HEPs. These coefficients were considered very strong for ICC ≥0.9, strong for 0.75 ≤ ICC <0.9, moderate for 0.5 ≤ ICC <0.75, and weak for ICC <0.5. The agreement between the skin test (SPT/PTP) results (positive/negative) and symptoms per food allergen was calculated by the number of patients with a positive skin test (SPT/PTP) and positive FASS. Subsequently, agreement between a negative skin test (SPT/PTP) and the absence of FASS was calculated. Agreements were tested using an exact binomial test. Confidence intervals (CI) were calculated for these ratios, with a significance level of 0.01 (Bonferroni correction). Data management was carried out from Castor. All calculations are performed in R (version 4.2.1, June 23, 2022). Comparison of qualitative skin test (SPT and PTP test) results and measured sIgE results with FASS, were reported as sensitivity, specificity, positive predictive value (PPV), NPV, concordance, and accuracy.

## 3 RESULTS

### 3.1 Study population

Thirty‐six adult patients (mean age 40.7; range 22–68 years, male nine) with FASS were included. Thirty‐six patients (100%) reported one or more inhalant allergies: 24 (67%) to grass pollen, 34 (94%) to birch pollen, 15 (42%) to house dust mites, and 19 (53%) to pets. Thirty‐tree (92%) patients reported the use of any anti‐allergic medication. Of the study group, 32 (89%) reported the use of anti‐histamines, 12 (33%) reported the use of a corticosteroid nasal spray, five (14%) reported the use of antihistamine eye drops, and nine (25%) reported the use of asthma medication. Eight (22%) of the patients needed to carry rescue medication (adrenalin) prescript due to another/additional allergy with more severe symptoms beyond OAS. All 36 (100%) of the patients reported food allergic symptoms, 34 (94%) reported OAS with or without GI symptoms, 12 (33%) reported a skin reaction, and 16 (44%) reported respiratory symptoms after ingestion of the specific food allergen. In Table 1, all patient characteristics are summarized.

**TABLE 1:** Patient characteristics.

Patient characteristics (*n* = 36)	
Female/male	27/9
Mean age/range (years)	40.7/22‐68

	*n* (%)
Inhalant allergy	36 (100)
Grass pollen	24 (67)
Birch pollen	34 (94)
House dust mite	15 (42)
Pets	19 (53)
Medication used	33 (92)
Anti‐histamines	32 (89)
Corticosteroid nasal spray	12 (33)
Antihistamine eye drops	5 (14)
Asthma medication	9 (25)
Adrenaline	8 (22)
None	3 (8)
Food allergy specific symptoms	36 (100)
GI + OAS	34 (94)
Skin	12 (33)
Respiratory	16 (44)

Abbreviations: GI, gastro‐intestinal symptoms; n, number; NA, not applicable; OAS, oral allergy symptoms; respiratory, respiratory symptoms; skin, skin symptoms.

The total number of patients who experienced symptoms after ingestion of the specific food allergen were: 27 (75%) for apple, 19 (53%) for kiwi, 16 (45%) for peach, nine (25%) for tomato, and eight (22%) for carrot. The total number of patients consuming the specific food allergen without any symptoms was nine (25%) for apple, 14 (39%) for kiwi, 13 (36%) for peach, 25 (69%) for tomato, and 22 (61%) for carrot. Eighteen skin tests were administered to patients who were unable to recall experiencing symptoms after consuming the specific food allergen. This lack of recollection was due to their adherence to a strict long‐term diet free from the food allergen, often based on a past positive sIgE result during routine testing. Results of FASS gathered from the questionnaire ever experienced after consumption of the specific food allergen are shown in Table 2.

**TABLE 2:** Symptoms per food allergen.

Symptoms per food allergen (*n* = 36)					
	Apple	Kiwi	Peach	Tomato	Carrot
	*n* (%)				
Symptoms per food allergen	27 (75)	19 (53)	16 (45)	9 (25)	8 (22)
Consuming/no symptoms	9 (25)	14 (39)	13 (36)	25 (69)	22 (61)
NA (strict diet)	0	3 (8)	7 (19)	2 (6)	6 (17)
Symptoms					
GI/OAS	18 (50)	11 (31)	10 (28)	8 (22)	4 (11)
Skin	0	0	0	0	0
Respiratory	0	0	0	0	0
GI/OAS + Skin	2 (6)	0	0	0	0
GI/OAS + Respiratory	4 (11)	6 (17)	3 (8)	0	1 (3)
GI/OAS + Skin + Respiratory	3 (8)	2 (6)	3 (8)	1 (3)	3 (8)
Skin + Respiratory	0	0	0	0	0

Abbreviations: GI, gastro‐intestinal symptoms; n, number; NA, not applicable; OAS, oral allergy symptoms; respiratory, respiratory symptoms; skin, skin symptoms.

### 3.2 Skin prick tests and prick‐to‐prick tests

In total, three hundred and sixty skin tests, 180 SPTs and 180 PTP tests, were performed using the five included foods allergens in 36 patients. The mean HEP index SPT versus PTP was; 0.86 versus 0.94 for apple, 0.35 versus 0.65 for kiwi, 1.10 versus 1.14 for peach, 0.20 versus 0.22 for tomato, and 0.43 equal for carrot. The ICCs between the SPT and PTP test results for apple and kiwi were moderate (0.72 and 0.71 resp.), strong for peach and tomato (0.75 and 0.89 resp.), and very strong for carrot (0.94). The HEP results of both diagnostic skin tests, ICC with 95% CI, *p*‐value of ICC, and strength of ICC of the five food allergens are shown in Table 3.

**TABLE 3:** Skin test results per food allergen.

Skin test results per food allergen (*n* = 36)						
		Apple	Kiwi	Peach	Tomato	Carrot
SPT	Positive ≥3 mm	31	22	31	11	25
	Mean HEP index	0.86	0.35	1.10	0.20	0.43
	Range HEP index	0‐3.92	0‐2.40	0‐5.20	0‐1.09	0‐2.75
PTP	Positive ≥3 mm	32	28	31	14	25
	Mean HEP index	0.94	0.65	1.14	0.22	0.43
	Range HEP index	0‐3.74	0‐4.10	0‐3.53	0‐1.52	0‐3.31
ICC		0.72	0.71	0.75	0.89	0.94
95% CI		0.52 < ICC < 0.85	0.35 < ICC < 0.87	0.56 < ICC < 0.87	0.80 < ICC < 0.94	0.89 < ICC < 0.97
*p*‐value		<0.0001	<0.0005	<0.0001	<0.0001	<0.0001
Strength		Moderate	Moderate	Strong	Strong	Very strong

Abbreviations: CI, confidence interval; HEP, histamine equivalent prick result; ICC, intra‐class correlation coefficient; mm, millimeter; n, number; PTP, prick‐to‐prick; SPT, skin prick test.

**FIGURE 1:**
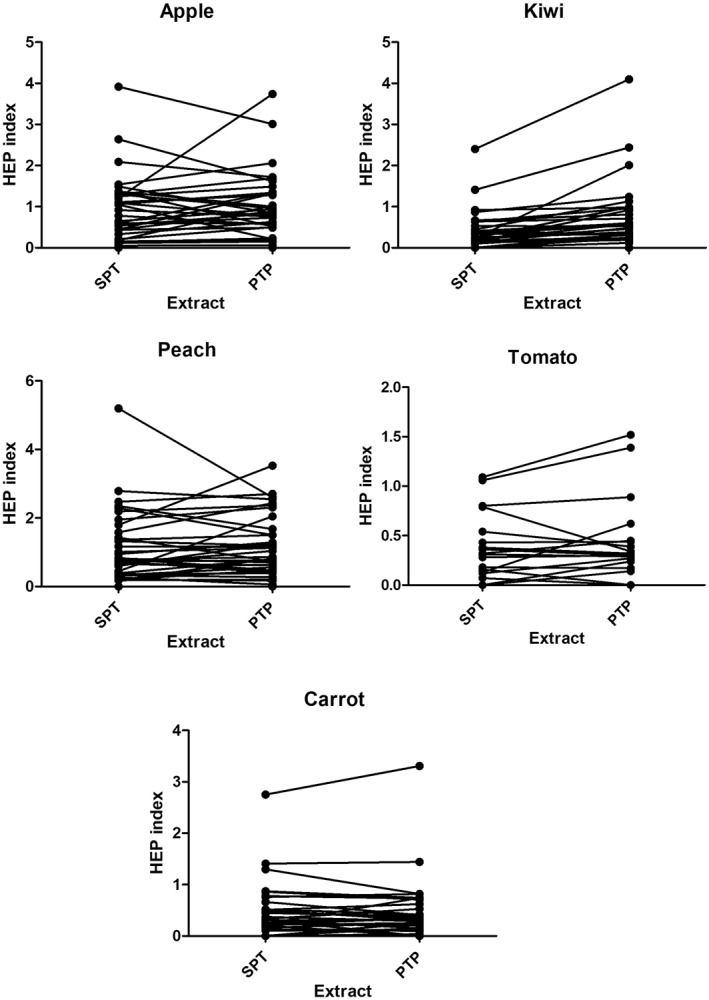
SPT‐PTP test HEP results of the five foods allergens.

The differences in the SPT‐PTP test HEP results of the five foods allergens are also depicted in Figure 1.

### 3.3 Accuracy of SPT‐HEP and PTP‐HEP results with FASS

**FIGURE 2:**
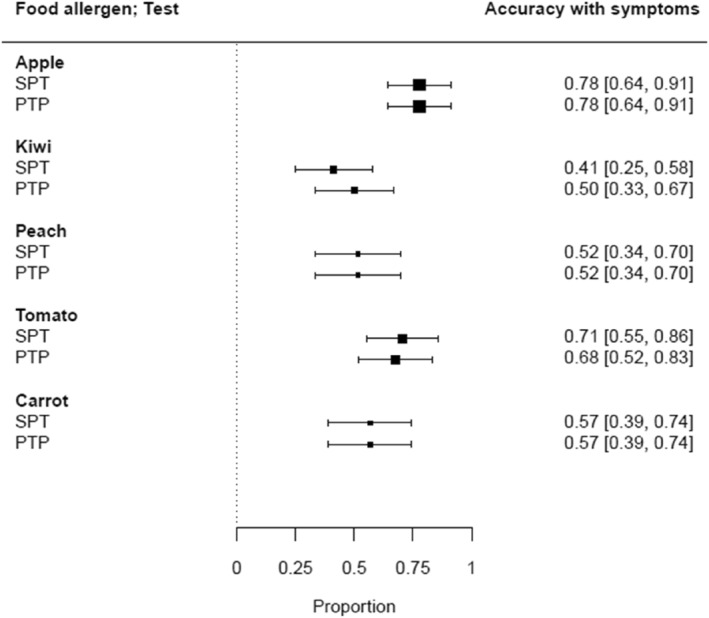
Accuracy of SPT‐PTP test HEP results in relation to FASS (proportion of agreement) and CI of the 5 food allergens. PTP, prick‐to‐prick; SPT, skin prick test.

We calculated an accuracy SPT versus PTP test with FASS obtained from the questionnaire of 0.78 equal for apple, 0.41 versus 0.50 for kiwi, 0.52 equal for peach, 0.71 versus 0.68 for tomato, and 0.57 equal for carrot. Accuracy of SPT‐PTP test HEP results in relation to FASS (proportion of agreement) and CI of all five food allergen extracts are shown in Figure 2.

### 3.4 Serum‐specific IgE measurements

Serum‐specific IgE levels of 35 participants were evaluated with the ALEX multiplex array (Macro Array Diagnostics, Vienna, Austria). The result was considered positive when >0.3 kUA/L. In the study group, specific IgE was positive in 23 for Mal d 1 (PR‐10 apple), zero for Mal d 3 (nsLTP apple), zero for Act d 2 (TLP kiwi), two for Act d 10 (nsLTP kiwi), one for Pru p 3 (nsLTP peach), one for Sola l (tomato extract), one for Sola l 6 (nsLTP tomato), nine for Dau c (carrot extract), and nine for Dau c 1 (PR‐10 carrot).

### 3.5 Accuracy of sensitization measurements in relation to FASS

Sensitivity and specificity measurements as well as the PPV, NPV, concordance, and accuracy of SPT and PTP test HEP results in comparison to FASS were obtained. The sensitivity of the SPT versus PTP test HEP results in comparison to FASS was equal for apple; 0.93, peach; 0.81, and carrot; 1.00, and comparable for kiwi; 0.50 versus 0.70 ) and tomato; 0.44 versus 0.56. The specificity of SPT versus PTP test HEP results in comparison to FASS was equal for apple; 0.33, peach; 0.15, and carrot; 0.41, and comparable for kiwi; 0.29 versus 0.21 resp., and tomato; 0.80 versus 0.72 resp. The mean accuracy of the SPT and PTP test HEP results of the five tested foods allergens was 0.60 versus 0.61. Sensitivity, specificity, PPV, NPV, concordance, and accuracy of sensitization measurements in relation to FASS are shown in Table 4.

**TABLE 4:** Accuracy of sensitization measurements in relation to FASS.

Accuracy of sensitization measurements in relation to FASS							
Allergen		Sensitivity	Specificity	PPV	NPV	Concordance	Accuracy
Apple	SPT	0.93	0.33	0.81	0.60	0.69	0.78
	PTP	0.93	0.33	0.81	0.60	0.69	0.78
	Mal d 1^a^	0.70	0.50	0.83	0.33	0.54	0.66
	Mal d 3^b^	0	1	NA	0.23	0	0.23
Kiwi	SPT	0.50	0.29	0.50	0.29	0.29	0.41
	PTP	0.70	0.21	0.56	0.33	0.41	0.50
	Act d 2^c^	0	1	NA	0.39	0	0.39
	Act d 10^b^	0.05	0.92	0.50	0.39	0.03	0.39
Peach	SPT	0.81	0.15	0.54	0.40	0.45	0.52
	PTP	0.81	0.15	0.54	0.40	0.45	0.52
	Pru p 1^a^	NA	NA	NA	NA	NA	NA
	Pru p 3^b^	0	0.92	0	0.44	0	0.43
Tomato	SPT	0.44	0.80	0.44	0.80	0.12	0.71
	PTP	0.56	0.72	0.42	0.82	0.15	0.68
	Sola l^d^	0.11	1	1	0.75	0.03	0.76
	Sola l 6^b^	0	0.96	0	0.72	0	0.70
Carrot	SPT	1	0.41	0.38	1	0.27	0.57
	PTP	1	0.41	0.38	1	0.27	0.57
	Dau c^d^	0.50	0.76	0.44	0.80	0.14	0.69
	Dau c 1^a^	0.50	0.76	0.44	0.80	0.14	0.69

Abbreviations: FASS, food allergen specific symptoms; NPV, negative predictive value; PPV, positive predictive value; PTP, prick‐to‐prick; sIgE, serum‐specific immunoglobulin E; SPT, skin prick test.

^a^ PR‐10, Pathogenesis Related protein 10.

^b^ ns‐LTP, non‐specific Lipid Transfer Protein.

^c^ TLP, Thaumatin‐Like Protein.

^d^ Extract Allergy Explorer (ALEX).

## 4 DISCUSSION

In this study, we investigated the comparison of SPT results performed using FJFV, and PTP test results performed using FFV. De Jong investigated in 2004 the reproducibility and stability of frozen aliquots of peach juice used in SPTs, and found no significant differences between fresh, 3 months, and 6 months old extracts.^12^ Garriga et al. described in 2010 the use of frozen aliquots of fresh fruits, in comparison to available commercial extracts and fresh fruit, as a good solution to perform SPTs at any time.^1^ In our in 2022 published first HoMaFa study, we compared the use of homemade extracts of apple and peach, with commercially available extracts.^16^

In the current study, comparison between SPT and PTP tests resulted in a very strong correlation for carrot, a strong correlation for peach and tomato, and a moderate correlation for apple and kiwi. These differences can be due to different methods applied to obtain the fruit extracts. Another aspect could be due to the rapid degradation of less stable allergens when being extracted and exposed to a different pH environment. Similarly, freezing and thawing could also destroy some of the food allergens in fruits such as apple and kiwifruit. We found comparable sensitivity and specificity between both test methods for apple, peach, tomato, and carrot. Kiwi SPT versus PTP test results showed some discrepancies in mean HEP index, range HEP index, sensitivity, and specificity. The number of positive SPT versus PTP test was 22 versus 28 resp, and mean HEP values showed almost double allergen‐induced wheal size in favor of the PTP test. This could be a consequence of the fact that by performing the PTP test, we chose to prick the needle into the fresh unpeeled kiwi near the stalk just under the fruit peel, where most proteins are located. For the SPT with frozen juice, the kiwi juice was obtained from the whole kiwi. Vlieg Boerstra et al. described the differences in apple PTP test results, caused by unequal distribution of Mal d one over the apple.^17^ Zivanovic et al. investigated in 2017 kiwi allergy in children,^18^ and recommend an oral food challenge if SPT and sIgE results did not correspond to the history.

Overall, for many years the availability of commercial food allergen extracts has been limited and even declining. Moreover, the available extracts are not standardized or contain insufficient quantities of the allergen, leading to the need for alternative methods for the extraction of food allergens. We recommend the use of FJFV. The juice was stored in small aliquots at −20°C, and could easily and quickly be defrosted before single use in SPTs. This enables the allergist to perform SPTs throughout the year, without limitations of the availability of seasonal fruits and vegetables.

There are some limitations to our study. One limitation is that we included 36 patients only, of which all 36 reported FASS in the history. However, the power of ICC calculations was >0.8 for all tested food allergens. In spite of this small dataset, we were able to demonstrate moderate to very strong correlations between both skin test methods. Another limitation is that 34 out of 36 patients ‐all selected in a general hospital‐ reported an inhalant allergy to birch pollen. Consequently, the majority of food allergic reactions may be based on cross‐reactivity between Bet v 1 and epitopes from different fruits such as apple (Mal d 1) and peach (Pru p 1).^3^ However, this patient group is particularly suitable for testing sensitization to fruits and vegetables. For that reason, in this particular group, we measured food allergen components for example, PR‐10 and nsLTP components by ALEX. Finally, we did not calculate the sensitivity, specificity, PPV and NPV against the DBPCFC. No oral provocation test was performed in this study. Subsequently, whether frozen apple, kiwi, peach, tomato, or carrot can be tolerated by the patient is unknown. Comparing SPT results with a food allergy‐focused clinical diet history questionnaire must be seen as a first step in the diagnosis of a food allergy.^19^ Although the use of the DBPCFC presumably will change the sensitivity and specificity calculations, the main goal of this study was to compare the SPT and PTP test results. Using a symptom‐based questionnaire as a reference allowed us to estimate the correlation between both test methods.

## 5 CONCLUSIONS

In this study, we demonstrate that SPT performed with FJFV of apple, kiwi, peach, tomato, and carrot is a valid method in the diagnosis of IgE‐mediated food allergy, in comparison with the PTP test performed with FFV. These results enable the clinician to test seasonal fruits or vegetables at any time of the year. We recommend further studies with homemade extracts of fruits and vegetables in another population, for example, children and patients with a primary food allergy, and to extend tests with other fruits and vegetables. Further, we recommend developing and validating educational tools on how to produce and use suitable and reproducible homemade food allergen extracts. These tools will increase the establishment of vertical and horizontal networks between Academic Centers of excellence, allergy specialists, and primary health care practitioners.^20^ These developments will increase the knowledge, quality, and use of homemade food allergen extracts, and might be one step forward in the complex diagnosis of an IgE‐mediated food allergy.

## AUTHOR CONTRIBUTIONS

**Severina Terlouw**: Conceptualization; data curation; formal analysis; investigation; methodology; project administration; resources; software; validation; visualization; writing ‐ original draft; writing ‐ review & editing. **Frank E. van Boven**: Data curation; formal analysis; software; validation; writing ‐ review & editing. **Monika Borsboom‐van Zonneveld**: Investigation; writing ‐ review & editing. **Tineke de Graaf‐in ’t Veld**: Investigation. **Roy Gerth van Wijk**: Supervision; writing ‐ review & editing. **Paul L. A. van Daele**: Supervision; writing ‐ review & editing. **Maurits S. van Maaren**: Writing ‐ review & editing. **Jac H. S. A. M. Kuijpers**: Formal analysis. **Sharon Veenbergen**: Formal analysis; writing ‐ review & editing. **Nicolette W. de Jong**: Formal analysis; methodology; software; supervision; validation; writing ‐ review & editing.

## CONFLICT OF INTEREST STATEMENT

The authors declare no conflicts of interest.

## Institutional Review Board Statement

Medical Ethical approval was obtained for this study at 27‐12‐2022, trial number MEC‐2022‐0716, NL81413.078.22. The study was conducted in accordance with the Declaration of Helsinki, and approved by the Ethics Committee of the Erasmus Medical Centre (protocol code HoMaFA‐2 study; MEC‐2022‐0716, NL81413.078.22; date of approval 27‐12‐2022).

## Informed Consent Statement

Informed consent was obtained from all subjects involved in the study. Written informed consent was obtained from all patients to publish this paper.

## Data Availability

The original database is not available online.
